# Two Structurally Different Dienelactone Hydrolases (TfdEI and TfdEII) from *Cupriavidus necator* JMP134 Plasmid pJP4 Catalyse *Cis*- and *Trans*-Dienelactones with Similar Efficiency

**DOI:** 10.1371/journal.pone.0101801

**Published:** 2014-07-23

**Authors:** Ajit Kumar, Balakrishna Pillay, Ademola O. Olaniran

**Affiliations:** Discipline of Microbiology, School of Life Sciences, College of Agriculture, Engineering and Science, University of KwaZulu-Natal (Westville Campus), Durban, Republic of South Africa; Russian Academy of Sciences, Institute for Biological Instrumentation, Russian Federation

## Abstract

In this study, dienelactone hydrolases (TfdEI and TfdEII) located on plasmid pJP4 of *Cupriavidus necator* JMP134 were cloned, purified, characterized and three dimensional structures were predicted. *tfdEI* and *tfdEII* genes were cloned into pET21b vector and expressed in *E. coli* BL21(DE3). The enzymes were purified by applying ultra-membrane filtration, anion-exchange QFF and gel-filtration columns. The enzyme activity was determined by using *cis*-dienelactone. The three-dimensional structure of enzymes was predicted using SWISS-MODEL workspace and the biophysical properties were determined on ExPASy server. Both TfdEI and TfdEII (M_r_ 25 kDa) exhibited optimum activity at 37°C and pH 7.0. The enzymes retained approximately 50% of their activity after 1 h of incubation at 50°C and showed high stability against denaturing agents. The TfdEI and TfdEII hydrolysed *cis*-dienelactone at a rate of 0.258 and 0.182 µMs^−1^, with a K_m_ value of 87 µM and 305 µM, respectively. Also, TfdEI and TfdEII hydrolysed *trans*-dienelactone at a rate of 0.053 µMs^−1^ and 0.0766 µMs^−1^, with a K_m_ value of 84 µM and 178 µM, respectively. The TfdEI and TfdEII k_cat_/K_m_ ratios were 0.12 µM^−1^s^−1^and 0.13 µM^−1^s^−1^ and 0.216 µM^−1^s^−1^ and 0.094 µM^−1^s^−1^ for for *cis*- and *trans*-dienelactone, respectively. The k_cat_/K_m_ ratios for *cis*-dienelactone show that both enzymes catalyse the reaction with same efficiency even though K_m_ value differs significantly. This is the first report to characterize and compare reaction kinetics of purified TfdEI and TfdEII from *Cupriavidus necator* JMP134 and may be helpful for further exploration of their catalytic mechanisms.

## Introduction

Dienelactone hydrolase or TfdE (DLH, EC 3.1.1.45) is the fifth enzyme in the 2, 4-dichlorophenoxyacetic acid (2, 4-D) degradation pathway which converts chlorodienelactone to chloromaleylacetate and plays a crucial role in the degradation of chloroaromatic compounds in *Cupriavidus necator* JMP134 and other microorganisms. Dienelactone hydrolase from *Cupriavidus necator* JMP134 and *Pseudomonas* sp. strain B13 (renamed as *Pseudomonas knackmussii*), categorized as type III DLH's are active against both *cis*- and *trans*-dienelactones [1]–[2]. Other reported DLH (type I) from *Cupriavidus necator* 335, *Pseudomonas putida* RW10, and *Pseudomonas reinekei* MT1 hydrolyse only *trans*-dienelactone [1], [3]–[4], while type II DLHs from *Rhodococcus opacus* 1CP and *Pseudomonas* (renamed as *Burkhoderia*) *cepacia* possess *cis*-dienelactone hydrolysing activity [5]–[6]. A complete most recent classification of different dienelactone hydrolases is tabulated elsewhere [7].

The DLH from *Pseudomonas knackmussii* B13 is the most thoroughly investigated dienelactone hydrolase [8]. This protein belongs to the α/β hydrolase fold enzymes [9] and is active against both the *cis*- and *trans*-isomer of dienelactone [2]. Overexpression of the gene encoding *trans*-DLH in *Pseudomonas reinekei* MT1 in *E. coli* [10] revealed that *trans*-DLH belongs to a poorly characterized protein family of putative Zn^2+^ dependent hydrolases and that the activity could be modulated by the exchange of Zn^2+^ by Mn^2+^ in at least two of the three metal-binding sites [11].

The catalytic mechanism and crystal structure of DLH from *Pseudomonas* sp. strain B13 (*knackmussii*) has been studied in detail [9], [12]–[14]. This enzyme has a catalytic triad composed of Cys-His-Asp in the active site and the sequence around the cysteine is Gly-Tyr-Cys-Leu-Gly-Gly [12]. A thermo-stable wild type and cloned DLH from *S. solfataricus* were also reported to contain a typical catalytic triad, Cys-Asp-His, in their active site [15]. This enzyme displayed remarkable stability against high temperature and various denaturing chemicals. It was classified as *trans*-DLH (type 1) and also exhibited carboxylesterase activity by hydrolysing *p*-nitrophenyl (PNP) caprylate (C_8_) [15].

The *tfdEI* and *tfdEII* genes in *Cupriavidus necator* JMP134 are temporally expressed at a very low concentration, thus making their product very difficult to purify directly from *Cupriavidus necator* JMP134 cultures grown in 2, 4-D, 2, 4-dichlorophenol (2, 4-DCP), 4-fluorobenzoate or other chloroaromatic substrate. Therefore, it is necessary to clone the *tfdEI* and *tfdEII* encoding genes in an expression vector and purify in high quantity to fully characterize and understand the catalytic mechanism of substrate hydrolysis by TfdEI and TfdEII enzymes. *Cupriavidus necator* JMP134 plasmid pJP4 harbours two distantly located chlorocatechol degradation clusters *tfd-I* and *tfd-II* [16] where isofunctional genes *tfdEI* and *tfdEII* expresses two dienelactone hydrolases TfdEI and TfdEII, respectively. Therefore, it will be highly significant to clone, overexpress and purify these enzymes and compare their catalytic activity. The *tfdEI* and *tfdEII* genes are 705 bp and 708 bp (41.5% homology) long and translate for 234a.a. and 235a.a. (19.68% homology) respectively. However, TfdEII and its mutant were expressed in *E. coli* and their activity in crude extract was compared on *trans*-dienelactone and *cis*-dienelactone, but not compared with TfdEI [17]. Till date, DLHs activity was observed in the cell lysates of *Cupriavidus necator* JMP134. The enzymes were partially purified [17] and their importance in *Cupriavidus necator* JMP134 discussed [7], [17]–[20] but none of the enzymes were completely purified. In this study, we have cloned, overexpressed TfdEI and TfdEII in *E. coli* and purified them to homogeneity. The catalytic properties as well as predicted three dimensional structures of these DLH's were also determined.

## Materials and Methods

### Media and growth conditions

A loopful of glycerol stock culture of *Cupriavidus necator* MP134 (The strain was kindly gifted by Prof. Bernardo Gonzalez, Facultad de Ingenieria y Ciencias, Adolfo Ibanez University, Santiago, Chile) was streaked onto nutrient agar plates and incubated at 30°C for 24 h and a single colony was further inoculated into 10 ml nutrient broth and grown at 30°C for 241h. The culture was centrifuged at 10000 rpm for 5 min and the pelleted cells were used for plasmid pJP4 isolation using GeneJET Plasmid Miniprep Kit (Cat. No. K0502, Thermo Scientific, South Africa). SeaKem LE Agarose for DNA gels was purchased from Lonza, USA. All the reagents used were of analytical grade.

### Cloning, expression and purification of recombinant DLH

The *tfdEI* (NCBI GeneID: 2847423) and *tfdEII* (NCBI GeneID: 2847410) encoding genes were amplified from plasmid pJP4 of *Cupriavidus necator* MP134, using primers pairs 5′- CGCGGATCCAATGTTATCAGACGGCGTTGAG-3′ and 5′- CGCAAGCTTTTACGAGGGGTCCTTCAACATTGC-3′ and primer pair 5′- CGCGGATCCAATGTGCCACGACACCGC-3′ and 5′- CGCAAGCTTTCAGCCCCCCAACAAGGTCTC-3′, respectively. The primers were manually designed according to the *tfdEI* and *tfdEII* gene sequences obtained from National Centre for Biotechnology Information (NCBI, http://www.ncbi.nlm.nih.gov/nuccore/39777443). The restriction sites for *Bam*HI and *Hind*III (as underlined above) were inserted into forward and reverse primers, respectively. The concentration of reagents for each PCR reaction (50 µl) were as follows: Primers, 1 µM each; dNTPs, 200 µM; MgCl_2_, 1.5 mM, 10 ng of template DNA and 1 U of Phusion Hot Start II High-Fidelity DNA polymerase (Fermentas, Thermo Scientific, South Africa). The PCR conditions include: 95°C for 5 min (1-Cycle); 95°C for 1 min, 60°C for 1 min, 72°C for 1 min (30-cycles) and final extension for 5 min at 72°C. Subsequently, the obtained 705 bp (*tfdEI*) and 708 bp (*tfdEII*) fragments were ligated into an expression vector pET21b (Novagen, U.S.A.) using *Bam*HI and *Hind*III (Fermentas, Thermo Scientific, South Africa) restriction sites (Figure S1). The ligation products were then transformed individually into chemically competent *E. coli* DH5α cells (Invitrogen) (Figure S2). The ligated products pET21b.*tfdEI* and pET21b.*tfdEII* were isolated using a GeneJET Plasmid Miniprep Kit (Cat. No. K0502, Thermo Scientific, South Africa) and again transformed into chemically competent *E. coli* expression cells BL21 (DE3) (Figure S3). The *E. coli* expression cells BL21(DE3) were grown at 37°C in 1 litre LB medium containing 100 µg/ml ampicillin and induced with 1 mM IPTG at A_600nm_ = 0.6. The cells were harvested after a 16 h incubation period at 30°C by centrifugation at 10000 rpm for 10 min. About 5 g of wet cells were re-suspended in 10 ml of 50 mM Tris buffer (pH 9.0) and lysed on ice using Sonic Ruptor 400 Ultrasonicator (OMNI International) (6 cycles each for 15-sec pulse). The lysate was obtained by centrifuging the lysis solution at 10000 rpm for 10 min. Recombinant TfdEI and TfdEII enzymes were purified by ultra-membrane filtration followed by column chromatography. The cell lysate (10 ml) was first passed through Amicon Ultra-15 Centrifugal Filter Unit (MW cut off 50 kDa, cat. no. UFC905024) at 5000×*g*. The flow-through (10 ml) containing proteins below Mr of 50 kDa then loaded on to Amicon Ultra-15 Centrifugal Filter Unit (MW cut off 10 kDa, cat. no. UFC901024) and centrifuged at 5000×*g*. About 1 ml of concentrate was collected in an Eppendorf tube and the flow-through was thrown out. These steps helped remove proteins above Mr of 55 kDa and below Mr of 10 kDa along with concentrating the proteins above Mr of 10 kDa to 1 ml. The Amicon ultra-15 centrifugal filter units were purchased from EMD Millipore Corporation, Billerica, MA, USA. A 5 ml anion exchange QFF column was equilibrated with 5 column volumes (CV) (1-CV = 5 ml) of 20 mM Tris buffer (pH 9.0) and 2 mg (1 ml) of total protein was loaded onto it. The unbound proteins were washed with 5-CV of 20 mM Tris buffer (pH 9.0). The proteins bound to the matrix were eluted with 10 CV of a 0–1.0 M linear gradient of NaCl in 20 mM Tris buffer (pH 9.0). The eluted proteins were collected as 2 ml fractions by using AKTA purifier100-P950 automated fraction collector. The fractions showing *cis*-dienelactone hydrolysing activity (please see section 3.3 for *cis*-dienelactone hydrolysing activity assay) were pooled together and concentrated by using a new Amicon Ultra-15 Centrifugal Filter Unit (MW cut off 10 kDa). One ml of 0.5 mg total protein then was loaded on a 53 ml ( = 1-CV) gel filtration column manually packed with Sephacryl HR100 matrix (purchased from Sigma-Aldrich, St. Louis, MO, USA) and pre-equilibrated with 2-CV of 20 mM Tris buffer (pH 9.0). The proteins were eluted with 2-CV and collected as 2 ml factions. The fractions showing *cis*-dienelactone hydrolysing activity were again pooled together and concentrated by using a new Amicon Ultra-15 Centrifugal Filter Unit (MW cut off 10 kDa). All the chromatography purification steps were performed using AKTA purifier100 machine (Amersham Pharmacia) at a flow rate of 5 ml/min for anion exchange and 0.5 ml/min for gel-filtration. At each purification step, the enzyme activity was measured by using substrate *cis*-dienelactone (ε_280_ = 17,000 M^−1^cm^−1^) [21]; a small volume (200 µl) of fractions was concentrated by acetone precipitation and loaded on 12% SDS-PAGE to confirm the purity and homogeneity of the proteins [22]. The protein quantification was performed using the method of Bradford [23].

### Dienelactone hydrolase assays

The standard enzyme assay was performed by incubating the physiological substrates, *cis*-dienelactone (kindly supplied by Prof. W. Reineke, Chemische Mikrobiologie Bergische Universität Wuppertal, Germany) and *trans*-dienelactone (kindly provided by Prof. Tore Benneche, University of Oslo, Norway) with the enzymes (final enzyme concentration in the reaction mixture 0.5 µM) and determined spectrophotometrically by measuring the decrease in substrate concentration at 280 nm. Substrate solution was prepared by mixing 0.1 ml (5 µM final concentration) of *cis*-dienelactone or *trans*-dienelactone (ε_280_ = 17,000 M^−1^cm^−1^ and ε_280_ = 15,625 M^−1^cm^−1^ respectively) with 0.8 ml of Tris-Cl buffer (pH = 7) in a quartz cuvette in Shimadzu UV-1800 UV-Vis Spectrophotometer fitted with temperature controller CPS-240A unit set at 37°C. After 1 min, 0.1 ml enzyme solution (0.5 µM) was added to the above substrate solution and incubated for 30 min [21]. To determine the rate of substrate hydrolysis by the enzymes, assays were performed by continuously monitoring the decrease in absorbance at 280 nm. The rate constants were calculated by fitting the data curves by non-linear regression equation.

### Initial kinetic analysis for the determination of K_m_, and K_cat_

The kinetic parameters for the substrate hydrolysis were determined by measuring the initial rate of enzymatic activity. The Michaelis-Menten constant (K_m_) was determined from the Lineweaver-Burk plot by applying the Michaelis-Menten equation. For the Lineweaver–Burk analysis, enzyme (0.5 µM) was incubated with *cis*- dienelactone (1 µM–1000 µM) and *trans*-dienelactone (1 µM–1000 µM) at 37°C for 30 min. The reciprocals of rate of substrate hydrolysis (1/V) were plotted against the reciprocals of the substrate concentrations (1/[S]), and the K_m_ values were determined by fitting the resulting data using ORIGIN 8 pro (evaluation version). V_max_ was also determined from the Lineweaver-Burk plot. The catalytic constant of the enzyme substrate reaction (K_cat_), also referred to as the turnover number, represents the number of reactions catalysed per unit time by each active site; was determined by the equation, K_cat_ = V_max_/[E]_t_, where V_max_ is the maximum velocity and [E]_t_, is the total enzyme concentration. Catalytic efficiency was calculated by the equation, K_cat_/K_m_.

### Carboxylesterase activity assay

The carboxylesterase activity assay was conducted spectrophotometrically using *p*-nitrophenyl (PNP) caprylate (C_8_) (Sigma-Aldrich, USA) by continuously monitoring the increase in absorbance at 405 nm due to the release of *p*-nitrophenol as previously described [15], [24]–[25]. The enzyme reaction was initiated by the addition of 0.1 ml of freshly prepared and pre-warmed PNP-caprylate solution (5 mM) as a substrate to 0.1 ml enzyme (0.5 µM) and 0.8 ml of pre-warmed 50 mM Tris-Cl (pH 7.0) at 37°C.

### Catalytic properties TfdEI and TfdEII

The optimum pH and temperature for TfdEI and TfdEII activity were determined by standard enzyme assays using, *cis*-dienelactone as the substrate. The effect of pH on the enzyme activity was examined at 37°C over a pH range of 3.0 to 9.0 using sodium citrate (pH 3.0 to 6.0), sodium phosphate (pH 6.0 to 8.0), and Tris-Cl (pH 8.0 to 9.0) buffers (50 mM). The effect of temperature on the enzyme activity was investigated at the optimum pH (Tris-Cl buffer pH 7.0) in the temperature range of 25°C to 70°C. The thermal stability of the enzyme was examined using the standard enzyme assay after incubation of the enzyme (0.5 µM) for designated time periods (0 to 5 h) at three different temperatures (30°C, 37°C and 50°C). The stability of the enzyme in the presence of 1% and 5% (w/v) detergents; Tween 80, Triton X-100, SDS; and denaturing agent, urea (4 M and 8 M) was investigated by measuring the residual enzyme activity after incubation of the enzyme (0.5 µM) with each compound for 60 min at 37°C. Blank samples were prepared by incubation of the compounds with the buffer solution instead of the enzyme under the same conditions. Control experiments were performed in the absence of each compound under the same conditions. To investigate the effect of divalent cations on the dienelactone hydrolase activity, 1 mM and 5 mM of each divalent cation (CaCl_2_, CuSO_4_, FeSO_4_, MnCl_2_, MgCl_2_, and ZnSO_4_) was separately added to the enzyme (0.5 µM) and incubated for 1 h at 37°C. The effect of EDTA on the enzyme activity was measured by adding 1 mM or 5 mM EDTA to the enzyme (0.5 µM) and incubated for 60 min at 37°C. The residual activities were measured using the standard enzyme assay after incubation [10], [15].

### Template based structure prediction

The TfdEI (234a.a.) and TfdEII (235a.a.) were searched on NCBI protein database using key words “TfdEI and TfdEII”. Homology search was carried out using PSI-BLAST in NCBI (non-redundant) database using ORF (open reading frame) of the protein as query sequence. Biophysical properties of the protein were determined using ExPASy server. The local sequence alignment was carried out by ClustalW2 (http://www.ebi.ac.uk/Tools/msa/clustalw2/). Structure prediction of TfdEI and TfdEII, was carried out using SWISS-MODEL workspace (http://swissmodel.expasy.org/workspace/index.php?func=modelling_ simple1&userid=USERID&token=TOKEN) [26]–[28].

### Statistics

All the kinetic parameters were determined by fitting the data using software ORIGIN 8 pro. For the kinetic analysis and rate constant determinations, the assays were carried out in triplicate, and the average value was considered throughout this work. *P* values less than 0.05 were considered to indicate statistical significance.

## Results and Discussion

### Over expression and purification of TfdEI andTfdEII

**Figure 1:**
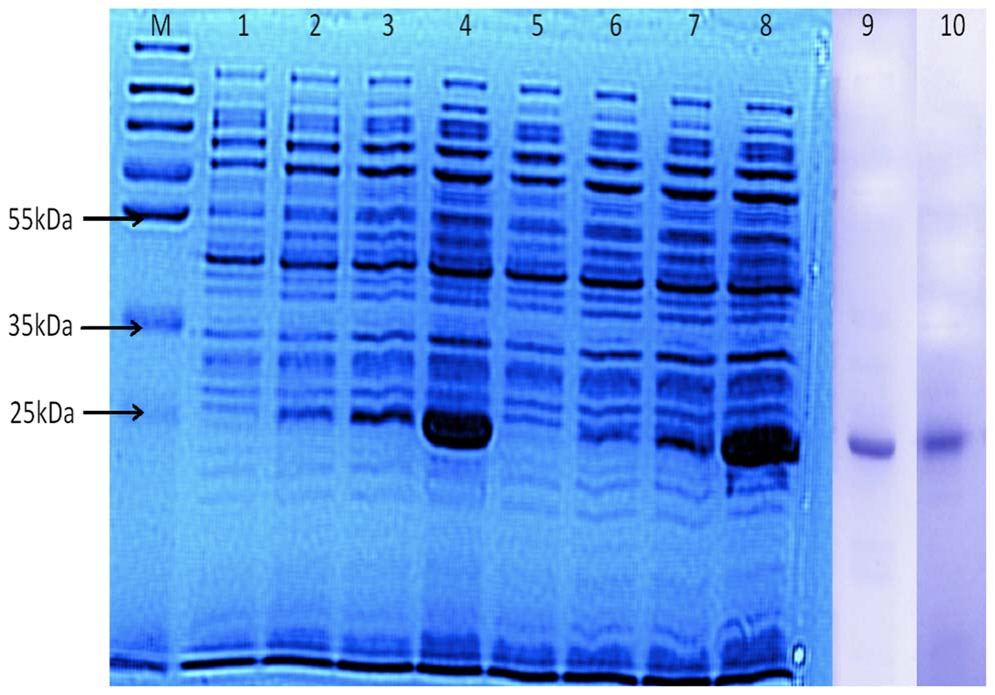
Expression and purification of TfdEI and TfdEII. Lane M shows protein markers, Lanes 1 and 5 show the un-induced lysate of TfdEI and TfdEII respectively. Lanes 2, 3 and 4 show the expression of TfdEI after 1 h, 5 h and 16 h respectively. Lanes 6, 7 and 8 show the expression of TfdEII after 1 h, 5 h and 16 h respectively. Lanes 9 and 10 show the purified TfdEI and TfdEII respectively.

*tfdEI* and *tfdEII* genes were inserted into the expression vector pET21b and successfully transformed in chemically competent cells of *E. coli* BL21 (DE3). Cell lysate of IPTG-induced *E. coli* Bl21 (DE3) exhibited the presence of prominent protein bands with an apparent Mr of about 25 kDa (Figure 1), which is in agreement with that deduced by the TfdEI and TfdEII gene product. The samples collected after ultrafiltration passed through 5 ml anion exchange QFF anion exchange column showed that fractions from A9–A13 hydrolysed *cis*-dienelactone (Figure S4 and Figure S5). The fractions pooled, concentrated and loaded on gel-filtration column showed that fraction A8–A12 hydrolysed *cis*-dienelactone (Figure S6 and Figure S7). Fractions pooled concentrated and loaded on 12% SDS-PAGE confirmed the purity and homogeneity of the protein. The results show that these enzymes are monomeric (Figure 1). The enzymes were purified to apparent homogeneity in four steps as shown in Table 1. TfdEI and TfdEII were purified to 125.5- and 131.6-fold, with a yield of 6.2% and 4.8%, respectively. Purified TfdEI and TfdEII showed a specific activity {defined as the enzyme activity (U/ml) divided by the total protein concentration (mg/ml) shown at the specific step of purification} of 857 U/mg protein and 708 U/mg protein respectively for the hydrolysis of the substrate, *cis*-dienelactone. TfdEII and its mutant expressed in *E. coli* crude extract exhibited specific activity of 2 and 2.6 U/mg on *trans*-dienelactone and *cis*-dienelactone, respectively, and indicate that this enzyme catalyse both substrate with similar efficiency [17]. A DLH was reported from the crude extract of *Pseudomonas* sp. Strain B13 with 24-fold purification (48% recovery) and specific activity of 65 U/mg [8]. DLH purified and reported from same strain *Pseudomonas* sp. strain B13 in other studies reported to show specific activity of 205.5 U/mg [2] and 58.8 U/mg [3]. A DLH from *Pseudomonas cepacia* was also purified with different specific activity of 217 U/mg [6]. Previous reports on the purification of DLH are from wild type organisms where DLH is expressed in low concentration. Therefore, complete recovery of the protein was not possible, which may be due to low specific activity observed. In this study, DLH's were purified after overexpression in *E. coli* strains, hence the high specific activity obtained even at low recovery of the proteins as indicated in table 1.

**Table 1:** Purification of TfdEI and TfdEII.

Purification Steps	Total protein (mg/ml)		Total activity (U/ml)		Specific activity (U/mg)		Yield (%)		Purification (fold)	
	TfdEI	TfdEII	TfdEI	TfdEII	TfdEI	TfdEII	TfdEI	TfdEII	TfdEI	TfdEII
Crude Cell extract	56	65	383	350	6.84	5.38	100	100	1	1
UM55 kDa	32	35	305	317	9.53	9.05	79	90	1.4	1.7
UM10 kDa	1.6	1.55	82	68	51.25	43.9	21.4	19.4	7.5	8.2
Anion Ex QFF	0.25	0.23	29	21	116	91	7.5	6	17	16.9
Sephacryl HR100	0.028	0.024	24	17	857	708	6.2	4.8	125.5	131.6

### Catalytic properties of TfdEI andTfdEII

**Figure 2:**
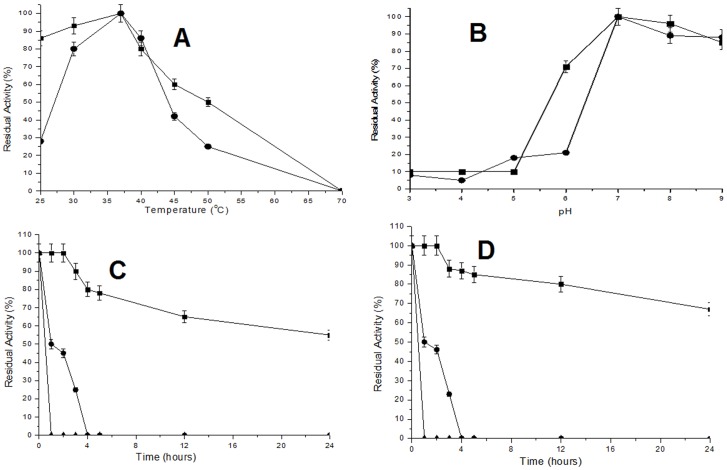
Optimum temperature, optimum pH and temperature stability of TfdEI and TfdEII. (**A**) Optimum temperature of TfdEI (▪) and TfdEII (•); (**B**) optimum pH of TfdEI (▪) and TfdEII (•);(**C**) temperature stability of TfdEI at 37°C (▪), 50°C (•) and 70°C (♦) and (**D**) temperature stability of TfdEII at 37°C (▪), 50°C (•) and 70°C (♦).

Both TfdEI and TfdEII displayed optimum activity at 37°C and pH 7.0 (Figure 2A and Figure 2B respectively). The other reported DLH's have exhibited optimum activity at 37°C [2], [3], [6], [8] while optimum pH is varying where DLH from *Pseudomonas* sp. Strain B13 showed optimal activity at pH 7.5 [2] and DLH from *Pseudomonas cepacia* exhibited optimal activity at pH 5.5 [6]. TfdEI and TfdEII retained approximately 50% of their activity after 1 h of incubation at 50°C (Figure 2C and Figure 2D respectively). Dienelactone hydrolase enzymes previously reported from mesophilic bacteria were not tested for their thermostability [2], [3], [6], [8]. In this study, TfdEI and TfdEII display moderate stability at mesophilic temperatures and in the presence of various compounds such as detergents, organic solvents, along with urea which normally affects the enzyme structure [29]. TfdEI and TfdEII showed high stability against Tween 80 and TritonX-100. TfdEII exhibited higher residual activity (70%) in the presence of 5% Tween 80 as compared to 1% Tween 80 (50% residual activity). This may be attributed to higher stability of the enzyme in the presence of 5% Tween 80 as compared to 1% Tween 80 as many studies have reported Tween 80 to increase enzyme stability and yield of proteins [30] The other dienelactone hydrolase from this class of enzymes are not reported to show stability against denaturing agents except a thermostable DLH from thermophilic archaea *Sulfolobus solfataricus* PI which has shown high thermo-stability and stability against denaturing agents [15], [24]–[25]. The stabilities of TfdEI and TfdEII against general protein-denaturing compounds such as SDS and urea obtained in this study indicate that strong hydrophobic interactions make up the stable core of these enzymes and that the enzymes may have a high surface hydrophobicity [31]–[32], which are attractive properties for their possible industrial and biochemical applications. Contrary to a report that activity of DLH from *Pseudomonas reinekei* MT1 is metal (especially Zn^2+^) dependent, [10], the absence of metal ions like Ca^2+^, Cu^2+^, Fe^2+^, Mn^2+^, Mg^2+^, and Zn^2+^ in the reaction did not have any effect on the activity of both DLHs (TfdEI and TfdEII) in this study, retaining 100% of their activities (Table 2). The observed lack of inhibition of activity of purified TfdEI and TfdEII in the presence of EDTA in this study is similar to that reported for DLH from *B. cepacia* (previous name: *Pseudomonas cepacia*) [3] and *Pseudomonas putida* strain 87 [33]. On the contrary, the activity of the trans-dienelactone hydrolase from *Pseudomonas reinekei* MT1 is significantly inhibited by EDTA [4], and the enzyme has been recently reported to be zinc-dependent [10]. Surprisingly, both TfdEI and TfdEII do not hydrolyse substrate *p*-nitrophenyl (PNP) caprylate (C_8_) which suggests the absence of carboxylesterase activity whereas previous study on the hydrolysis of *p*-nitrophenyl (PNP) caprylate (C_8_) shows that some dienelactone hydrolases exhibit carboxylesterase activity [15].

**Table 2:** Residual activity (%) of TfdEI and TfdEII in the presence of different reagents and metal ions.

Regents and metal ions	Concentration	Residual activity (%)	
		TfdEI	TfdEII
Tween 80	1%	95	50
	5%	95	70
Triton X100	1%	95	95
	5%	95	95
Urea	4 M	50	0
	8 M	0	0
SDS	1%	0	0
	5%	0	0
EDTA	1 mM	100	100
	5 mM	100	100
Ca^2+^	1 mM	100	100
	5 mM	100	100
Cu^2+^	1 mM	100	100
	5 mM	100	100
Fe^2+^	1 mM	100	100
	5 mM	100	100
Mn^2+^	1 mM	100	100
	5 mM	100	100
Mg^2+^	1 mM	100	100
	5 mM	100	100
Zn^2+^	1 mM	100	100
	5 mM	100	100

**Figure 3:**
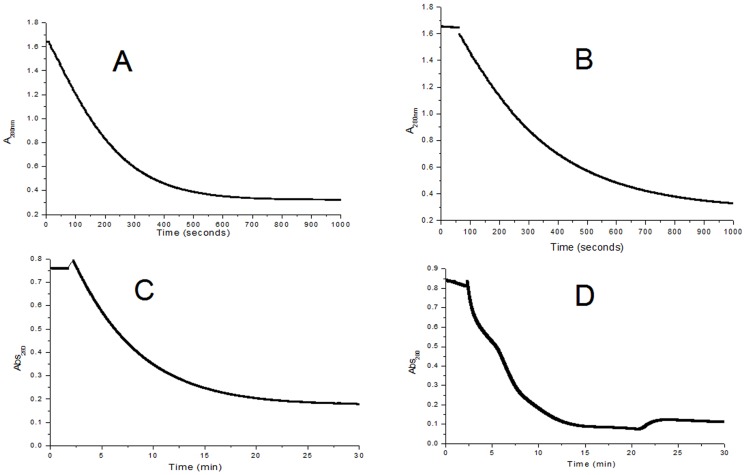
Reaction kinetics of TfdEI and TfdEII (0.5 µM). (**A**) Rate of reaction for the hydrolysis of *cis*-dienelactone by TfdEI; (**B**) Rate of reaction for the hydrolysis of *cis*-dienelactone by TfdEII; (**C**) Rate of reaction for the hydrolysis of *trans*-dienelactone by TfdEI and (**D**) Rate of reaction for the hydrolysis of *trans*-dienelactone by TfdEII.

**Figure 4:**
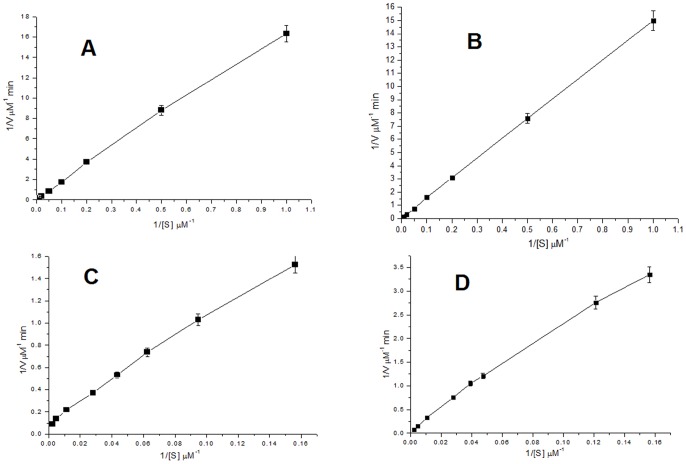
Lineweaver-Burk plot for TfdEI and TfdEII. (**A**) Lineweaver-Burk plot for the hydrolysis of *cis*-dienelactone by TfdEI; (**B**) Lineweaver-Burk plot for the hydrolysis of *cis*-dienelactone by TfdEII; (**C**) Lineweaver-Burk plot for the hydrolysis of *trans*-dienelactone by TfdEI and (**D**) Lineweaver-Burk plot for the hydrolysis of *trans*-dienelactone by TfdEII.

The TfdEI and TfdEII (0.5 µM) converted *cis*-dienelactone to maleylacetate at a rate of 0.258 and 0.182 µMs^−1^ (Figure 3A and Figure 3B respectively), with a K_m_ value of 87 µM and 305 µM respectively (Figure 4A and Figure 4B respectively). However, conversion of *trans*-dienelactone to maleylacetate occurred at a rate of 0.053 µMs^−1^ and 0.0766 µMs^−1^ (Figure 3C and Figure 3D respectively) which is 4.87-fold (TfdEI) and 2.38-fold (TfdEII) lower than the rate of *cis*-dienelactone conversion, with a K_m_ value of 84 µM and 178 µM, respectively (Figure 4C and Figure 4D respectively). The k_cat_/K_m_ ratios were found to be 0.12 µM^−1^s^−1^and 0.13 µM^−1^s^−1^ for *cis*-dienelactone and 0.216 µM^−1^s^−1^ and 0.094 µM^−1^s^−1^ for *trans*-dienelactone for TfdEI and TfdEII, respectively. The catalytic efficiency ratio 0.55 of *cis*/*trans* for TfdEI shows that *trans*-dienelactone is hydrolysed more efficiently than *cis* isomeric form of the substrate, while the *cis*/*trans* ratio of 1.38 obtained for TfdEII shows that *cis*-dienelactone is hydrolysed more efficiently than *trans*- isomeric form of the substrate. All the kinetic analysis values are summarized in table 3. DLH from *Pseudomonas* sp. Strain B13 hydrolyses both *cis*- (K_m_ = 400 µM) and *trans*- (K_m_ = 400 µM) isomers of dienelactones [2]. The k_cat_ observed with for each dienelactone was 1,800 min^−1^ [2] which is much higher than the values exhibited by the enzymes in this study (10.44 min^−1^ for TfdEI and 39.6 min^−1^ for TfdEII). However, TfdEI and TfdEII characterized from *Cupriavidus necator* JMP134 in this study catalyse *cis*-dienelactone more efficiently and *trans*-dienelactone less efficiently than the previously reported DLH's from *Pseudomonas* sp. Strain B13 [2] and *Pseudomonas cepacia* [6] as shown by K_m_ values. *Pseudomonas* sp. Strain B13 was shown to hydrolyse protoanemonin with a K_m_ value of 415 µM and *cis*-dienelactone with a K_m_ value of 381 µM [3] which are higher than that reported in this study. The cell free extract of *Cupriavidus necator* JMP134 shown to catalyse *trans*-dienelactone and *cis*-dienelactone with the K_m_ value of 100 µM and 140 µM, respectively, [7] which are comparable to the K_m_ values reported in this study.

**Table 3:** Kinetic properties of TfdEI and TfdEII (0.5 µM).

Kinetic Properties	*cis*-Dienelactone		*trans*-Dienelactone		*cis/trans* ratio	
	TfdEI	TfdEII	TfdEI	TfdEII	TfdEI	TfdEII
Rate (µM/sec)	0.26	0.18	0.05	0.08	5.20	2.25
K_m_ (µM)	87	305	84	178	1.04	1.71
V_max_(µM/min)	5.22	20	9.09	8.38	0.57	2.39
K_cat_(min^−1^) = V_max_/Et	10.44	39.60	18.18	16.74	0.57	2.37
Catalytic efficiency (K_cat_/K_m_) (µM^−1^s^−1^)	0.12	0.13	0.22	0.09	0.55	1.44

### Comparison of amino acid sequence homology

**Figure 5:**
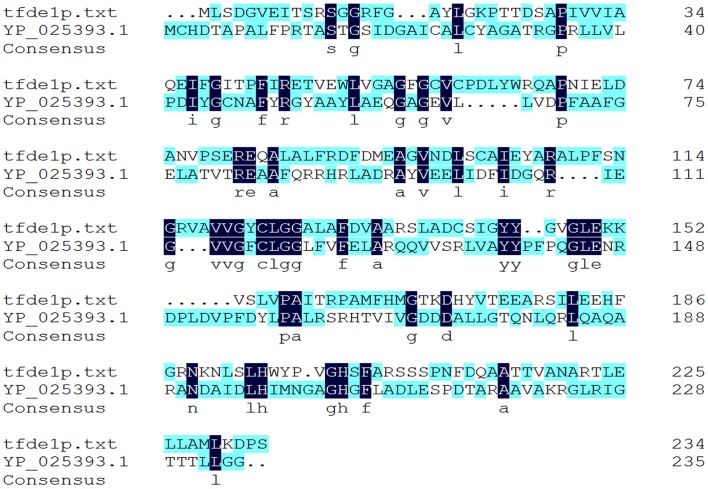
CLUSTAL O (1.1.0) multiple protein sequence alignment for TfdEI (shown as tfdeIp.txt) and TfdEII (shown as YP_025393.1). The amino acids highlighted with blue colour are the homolog amino acids. TfdEI Glu36, Arg81, and Arg206 in addition to Cys123, Asp171, and His 202 plays crucial role in catalysis while the corresponding amino acids in TfdEII are Asp42, Arg82, Cys117, Ala206, Asp173 and His 204.

**Figure 6:**
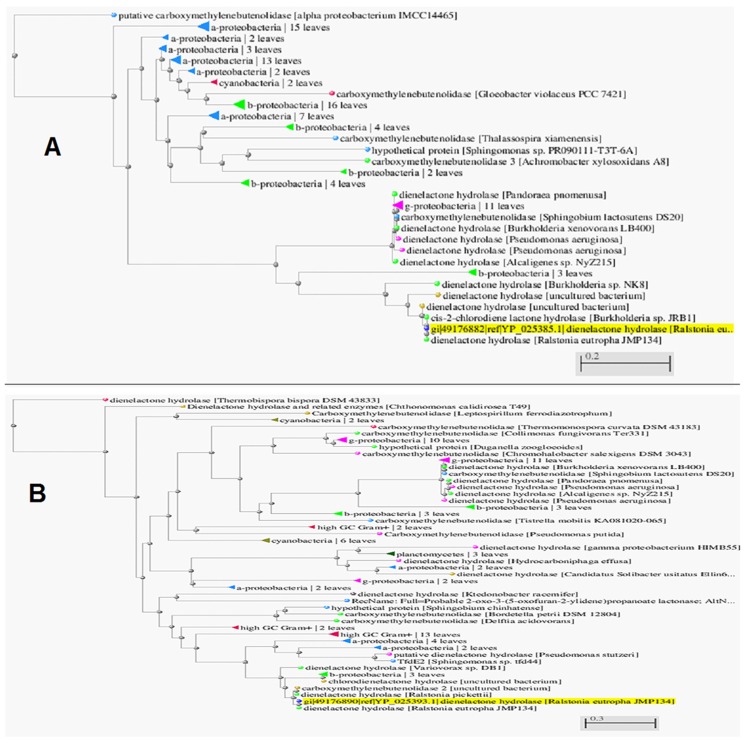
NCBI protein sequence blasted distance tree for TfdEI (A) and TfdEII (B). Highlighted with yellow colour are the DLH's in this study.

Homologs of the enzyme were searched using BLASTP in the NCBI (non-redundant) database [34]–[35]. TfdEI (YP_025385.1) and TfdEII (YP_025393.1) protein sequence homology shows that these enzymes have evolved very distinctly (Figure 5), with only 19% identity to each other at protein level. Results of the BLAST analysis of TfdEI and TfdEII protein sequence in NCBI are shown in Figure 6A and 6B, respectively. The highest similarity (81%) was found between TfdEI and DLH from *Burkholderia* sp. NK8. TfdEI showed 55% identity with DLH from *Pandoraea pnomenusa* and *Pseudomonus* sp. B13 while 53% identity with DLH from *Achromobacter xylosoxidans* A8 and *Bordetella* sp. IITR-02. TfdEI showed a conserved domain for dienelactone hydrolase and related enzymes (Secondary metabolites biosynthesis, transport, and catabolism) and α/β hydrolase family [9], [12]–[14], [36]. The highest similarity (86%) was found between TfdEII and DLH from *Variovorax* sp. DB1. TfdEII also shared 79% homology with DLH from *Delftia acidovorans* and *Achromobacter denitrificans*. The DLH from *Burkholderia cepacia* showed 78% identity with TfdEII. TfdEII also showed a conserved domain for the dienelactone hydrolase family and related enzymes (Secondary metabolites biosynthesis, transport, and catabolism) but unlike TfdEI, it does not show a conserved domain with α/β hydrolase family [36].

### Template based structure prediction and prediction of the catalytic mechanism

**Figure 7:**
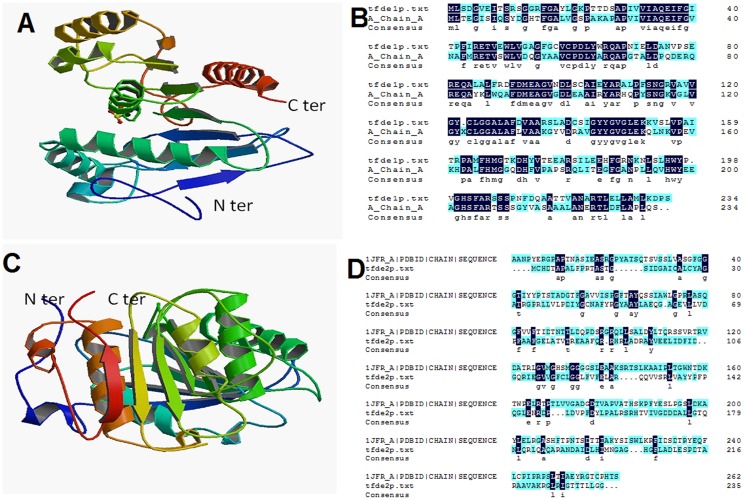
Predicted tertiary structure of TfdEI (A) deduced from PDB 1DIN and TfdEII (C) deduced from 1JFR-A. N- and C-terminal domains of the monomers are marked. Helices are presented in green and sheets in blue. The model was built by homology modelling at SWISS-MODEL workspace using TfdEI and TfdEII as the templates. (**B**) CLUSTAL O (1.1.0) multiple protein sequence alignment for TfdEI (shown as tfdeIp.txt) with 1DIN (shown as A_Chain_A). (**D**) CLUSTAL O (1.1.0) multiple protein sequence alignment for TfdEII (shown as tfdeIIp.txt) with 1JFR-A (1JFR_A |PDBID|CHAIN|SEQUENCE). The sequence homolog amino acids are highlighted in blue colour.

Homology modelling using 1.8 Å structure of DLH from *Pseudomonas* sp. B13 (PDB: 1DIN) as template revealed 55% resemblance of TfdEI with the model protein 1DIN (Figure 7A). All the residues involved in the catalytic site can be predicted from the structure as 1DIN and TfdEI are both classified as type III DLH's. TfdEI is active toward both *cis*- and *trans*-dienelactones like DLH from *P. knackmussii* (old name: *Pseudomonas sp*. Strain B13) [3]. Detailed study of the catalytic mechanism and crystal structure of the DLH from *P. knackmussii* revealed that Glu36, Arg81, and Arg206 in addition to Cys123, Asp171, and His 202, a catalytic triad, in the active site of the enzyme play a key role before and after substrate binding [37]. Structure prediction studies suggest that the corresponding amino acid residues in TfdEI are the same as those of *P. knackmussii*. It is possible that TfdEI hydrolyse dienelactone in the same manner as DLH from *P. knackmussii* since glutamic acid and the catalytic triad, Cys-Asp-His, are conserved in these two enzymes (Figure 7B). Predicted tertiary structure showed TfdEII sequences to be 17% identical to the model reference PDB:1JFR-A, which represents the crystal structure of a microbial homologue of mammalian platelet-activating factor, acetylhydrolases from *Streptomyces exfoliatus* lipase at 1.9 Å resolution (Figure 7D). The protein 1JFR-A is very distinct from TfdEII, therefore, the prediction of catalytic residues of TfdEII could not be done (Figure 7C). TfdEII seems to be more distantly evolved from TfdEI; however crystallographic study is required for better understanding of their catalytic mechanism.

### Predicted biophysical properties of the TfdEI and TfdEII

Biophysical properties of the DLH's were determined using ExPASy server [38]. The TfdEI and TfdEII show apparent Mr. of 25 kDa with a theoretical pI of 5.25 and 5.35 respectively (Table 4). The acidic nature of the proteins is congruent with the high content of acidic amino acids present than basic amino acids. The instability index of TfdEI is computed to be 48.95, thus classifying it as an unstable enzyme. This may be the reason for the requirement of a second cluster coding for a similar enzyme by *Cupriavidus necator* JMP134 [16]. On the other hand, TfdEII shows an instability index of TfdEI 37.94 and classified as stable. Hence, it may be argued that when TfdEI expression stops after a certain period of time, *tfdII* cluster expressed TfdEII which acts on the accumulated dienelactones to further hydrolyse them in the cell. However, time dependent expression of TfdEI and TfdEII using mRNA from cells induced by 2, 4-D or 2, 4-DCP needs to be investigated to confirm these results. TfdEI showed high content of aromatic amino acids (Trp, Tyr, Phe), resulting in a high extinction coefficient value (28420 M^−1^cm^−1^) as compared to TfdEII which showed an extinction coefficient value of 13410 M^−1^cm^−1^, which is about 50% lower than that of TfdEI (Table 4). The difference in the number of these amino acids in TfdEI and TfdEII therefore suggest that these two proteins are quite structurally different.

**Table 4:** Predicted biophysical properties of the TfdEI and TfdEII.

Property	Value	
	TfdEI	TfdEII
Number of amino acids	234	235
Molecular Mass (Da)	25427.90	25399.90
pI	5.25	5.35
Total negatively charged residues	27	27
Total positively charged residues	20	21
Extinction coefficient (if all Cys reduced)	28420 M^−1^ cm^−1^	13410 M^−1^ cm^−1^
Instability index	48.95 (classified as unstable)	37.94 (classified as stable)

Among all other *Cupriavidus* strains, DLHs from *Cupriavidus necator* JMP134 showed the highest specific activity when grown on 2, 4-D and 4-fluorobenzoic acid (4FB) [1]. Enzyme assays with cell extracts of different strains of *Alcaligenes* (now known as *Cupriavidus*) showed that DLH's with significantly different substrate specificities are expressed by 4FB-utilizing bacteria [1]. Extracts from *A. eutrophus* 335, *A. eutrophus* H16, *A. eutrophus* JMP222, and *Alcaligenes* strain A7 hydrolysed *trans*-dienelactone considerably faster than the *cis*-dienelactone (dienelactone hydrolase type I). An opposite preference for the two dienelactone isomers was observed in *P. cepacia* (dienelactone hydrolase type II). Extracts from *A. eutrophus* JMP134 (now known as *Cupriavidus necator* JMP134) hydrolysed *cis*- and *trans*-dienelactone at the same rate and order of magnitude. DLH isolated from *Pseudomonas* strain B13 exhibited substrate specificity similar to *Cupriavidus necator* JMP134 and can be thus classified as dienelactone hydrolase type III [6]–[8]. At substrate saturation (1 mM), DLH from *Pseudomonas* strain B13 hydrolyses both dienelactones at about the same rate, whereas at 0.1 mM, it hydrolyses the *trans*-dienelactone four times faster than the *cis*-dienelactone [1]. It could be speculated that an apparent type III dienelactone hydrolase activity is simulated by a mixture of type I and type II enzymes in the same extract of *Pseudomonas* strain B13 but this possibility was excluded since this enzyme was shown to hydrolyse both dienelactones, even after it had been purified to homogeneity [8]. The present study proves that this is also true for the pJP4-encoded DLH from *Cupriavidus necator* JMP134. The apparent presence of different types of DLH in *Cupriavidus necator* JMP134 and in its cured derivative *Cupriavidus necator* JMP222 implies that JMP134 possesses at least two DLH's, one of type III encoded by the plasmid pJP4 and another of type I encoded by the chromosome or by a megaplasmid [1], [39]. The type III enzyme was observed by Pieper et al. 1988 [40] and its gene was found to be localized on pJP4 [41]–[42]. At the time of DLH classification on the basis of a study by Schlomann et al. [1], it was not known that *Cupriavidus necator* JMP134 plasmid pJP4 encodes for two distantly located chlorocatechol degradation clusters *tfd-I* and *tfd-II* [16] where *tfdEI* and *tfdEII* genes that are not completely isofunctional express two DLHs. However, results from the present study prove that *tfdEI* and *tfdEII* are completely isofunctional. We propose that type III dienelactone hydrolase activity observed by Schlomann et al. [1] in the crude extract of *Cupriavidus necator* JMP134 might be due to both *tfdEI* and *tfdEII* gene products hydrolysing *trans*- or *cis*-dienelactone. Our studies on the hydrolysis of *trans*- and *cis*-dienelactone by purified TfdEI and TfdEII shows that both enzymes hydrolyze *cis*- and *trans*-dienelactone with the same efficiency which support the hypothesis by Schlomann et al. [1] that type I dienelactone hydrolase in JMP134 does not exist.

## Conclusions

Comparisons of the data from present study and previously reported studies conclude that TfdEI and TfdEII hydrolyse *cis*-dienelactone and *trans*-dienelactone with similar efficiency but may involve a different catalytic mechanism. Though, there are some reports on the function of *tfd-I* and *tfd-II gene* clusters [16]–[20], but it is still unclear why *Cupriavidus necator* JMP134 harbours two structurally different and distantly located DLHs on pJP4 which requires further investigation. Dienelactone hydrolases (TfdEI and TfdEII) converts chlorodienelactone to chloromaleylacetate and play a crucial role in the chloroaromatic compounds degradation pathway in *Cupriavidus necator* JMP134 and other microorganisms. Thus, this study may be helpful for further exploration of TfdEI and TfdEII catalytic mechanisms in order to fully understand the pathways involved in the degradation of chloroaromatic and other related compounds. The information can also be helpful for the effective use of *Cupriavidus necator* JMP134 as a possible bioaugmentation candidate for the degradation of 2, 4-dichlorophenoxyacetic acid.

## Supporting Information

Figure S1 **Amplification of 705 bp** ***tfdEI*** **(1) and 708 bp** ***tfdEII*** **(2) from plasmid pJP4 by using the primers with** ***Bam*** **Hi (Forward) and** ***Hind*** **III restriction sites and cut from the 1% agarose gel for the cloning the genes in pET21b.**
(PDF)

Figure S2 **Colony PCR of** ***E. coli*** **DH5α for the transformation of ligated genes in pET21b digested with** ***Bam*** **H1 and** ***Hind*** **III restriction enzymes using the primers mentioned in the study.**
(PDF)

Figure S3 **Colony PCR of** ***E. coli*** **Bl21 (DE3) for the transformation of ligated genes in pET21b digested with** ***Bam*** **H1 and** ***Hind*** **III restriction enzymes using the primers mentioned in the study.**
(PDF)

Figure S4 **Ion Exchange chromatogram for the purification of TfdEI. Fractions A9–A13 hydrolysed** ***cis*** **-dienelactone.**
(PDF)

Figure S5 **Ion Exchange chromatogram for the purification of TfdEII.** Fractions A9–A13 hydrolysed *cis*-dienelactone.
(PDF)

Figure S6 **Gel filtration chromatogram for the purification of TfdEI.** Fractions A8–A12 hydrolysed *cis*-dienelactone.
(PDF)

Figure S7 **Gel filtration chromatogram for the purification of TfdEII.** Fractions A8–A12 hydrolysed *cis*-dienelactone.
(PDF)
