# Supplementary figures and images for: Two Structurally Different Dienelactone Hydrolases (TfdEI and TfdEII) from Cupriavidus necator JMP134 Plasmid pJP4 Catalyse Cis- and Trans-Dienelactones with Similar Efficiency
Source: PLoS One. 2014 Jul 23;9(7):e101801. doi: 10.1371/journal.pone.0101801 (PMC4108320; doi:10.1371/journal.pone.0101801)

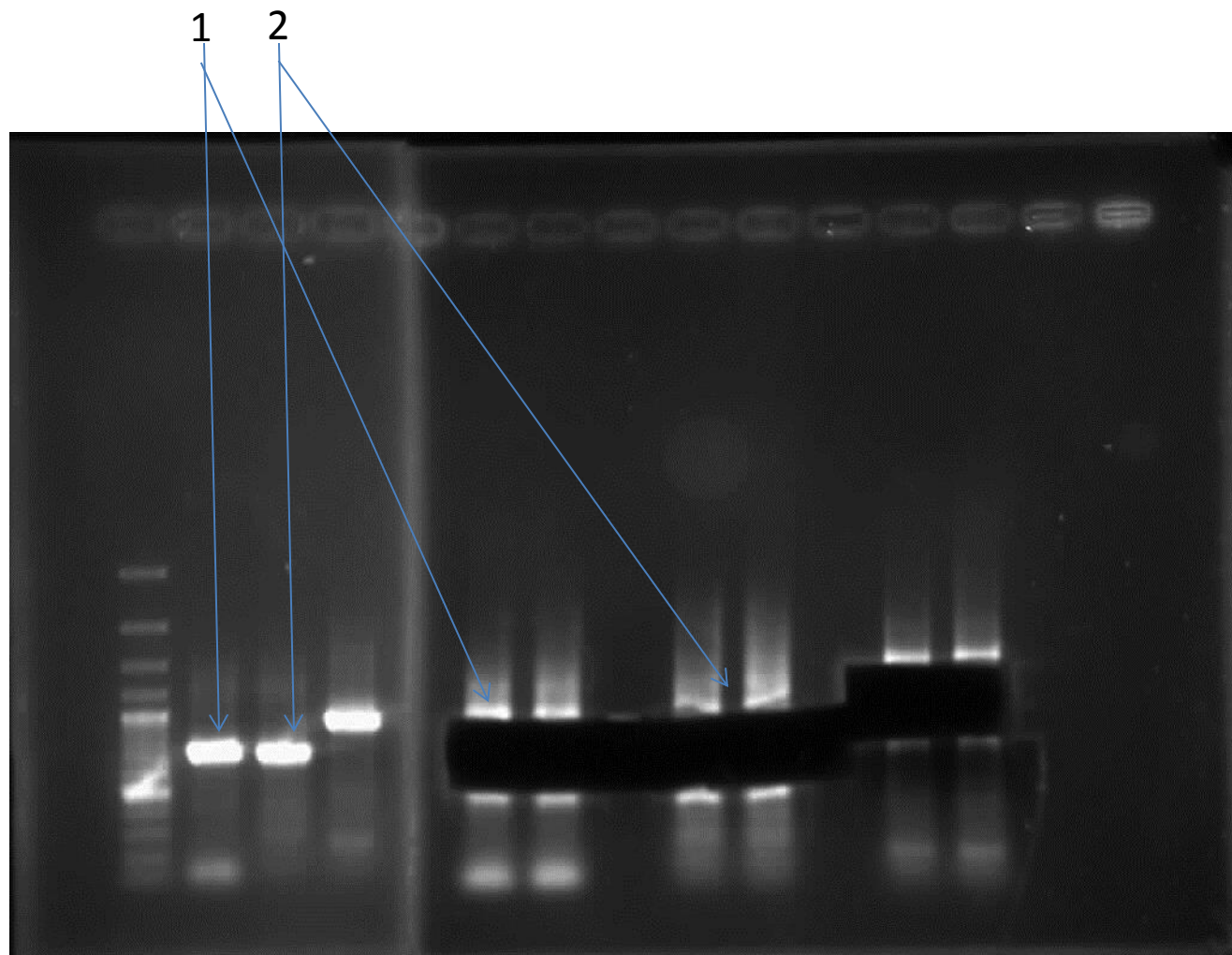

Supplement: Figure S1 — Amplification of 705 bp tfdEI (1) and 708 bp tfdEII (2) from plasmid pJP4 by using the primers with Bam Hi (Forward) and Hind III restriction sites and cut from the 1% agarose gel for the cloning the genes in pET21b. (PDF) [file pone.0101801.s001.pdf]

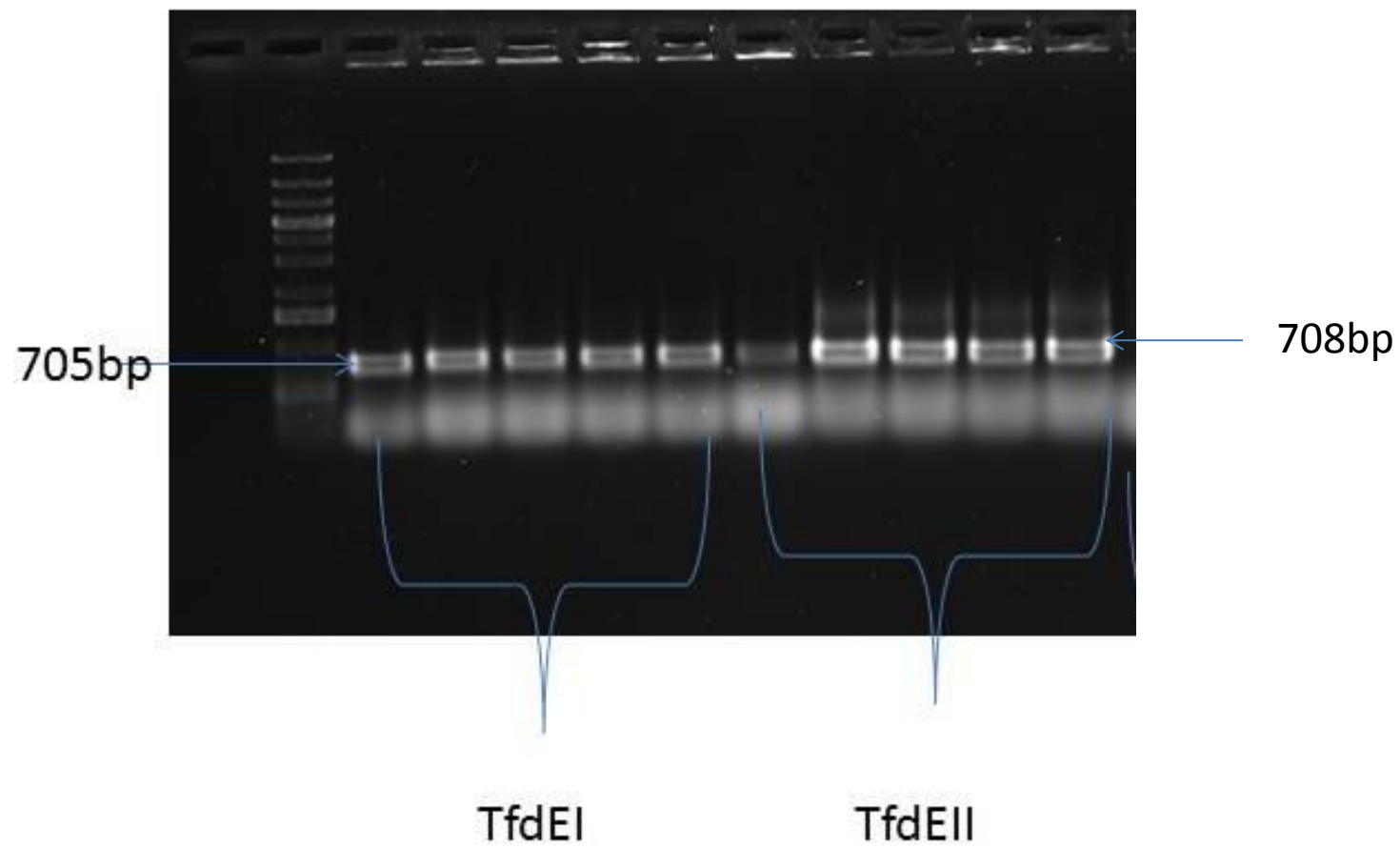

Supplement: Figure S2 — Colony PCR of E. coli DH5α for the transformation of ligated genes in pET21b digested with Bam H1 and Hind III restriction enzymes using the primers mentioned in the study. (PDF) [file pone.0101801.s002.pdf]

*tfdEI*

*tfdEII*

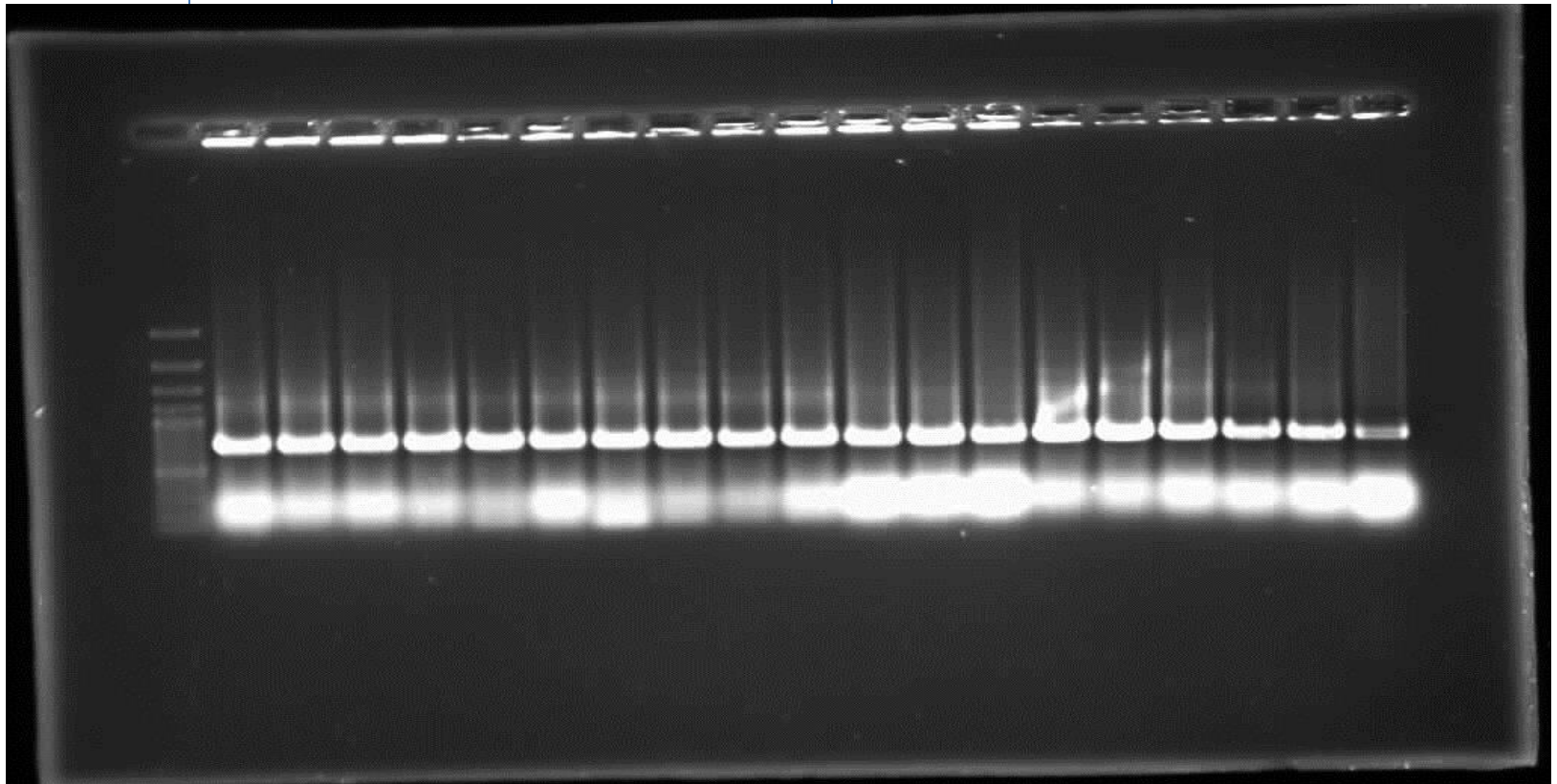

Supplement: Figure S3 — Colony PCR of E. coli Bl21 (DE3) for the transformation of ligated genes in pET21b digested with Bam H1 and Hind III restriction enzymes using the primers mentioned in the study. (PDF) [file pone.0101801.s003.pdf]

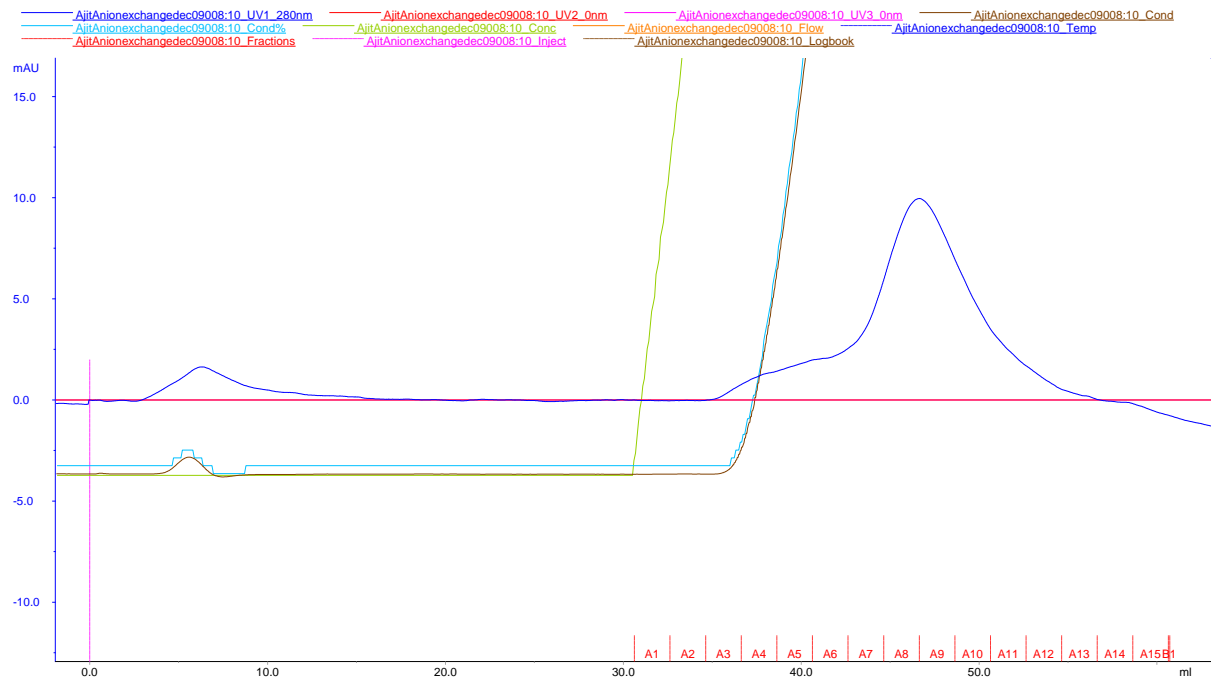

Supplement: Figure S4 — Ion Exchange chromatogram for the purification of TfdEI. Fractions A9–A13 hydrolysed cis -dienelactone. (PDF) [file pone.0101801.s004.pdf]

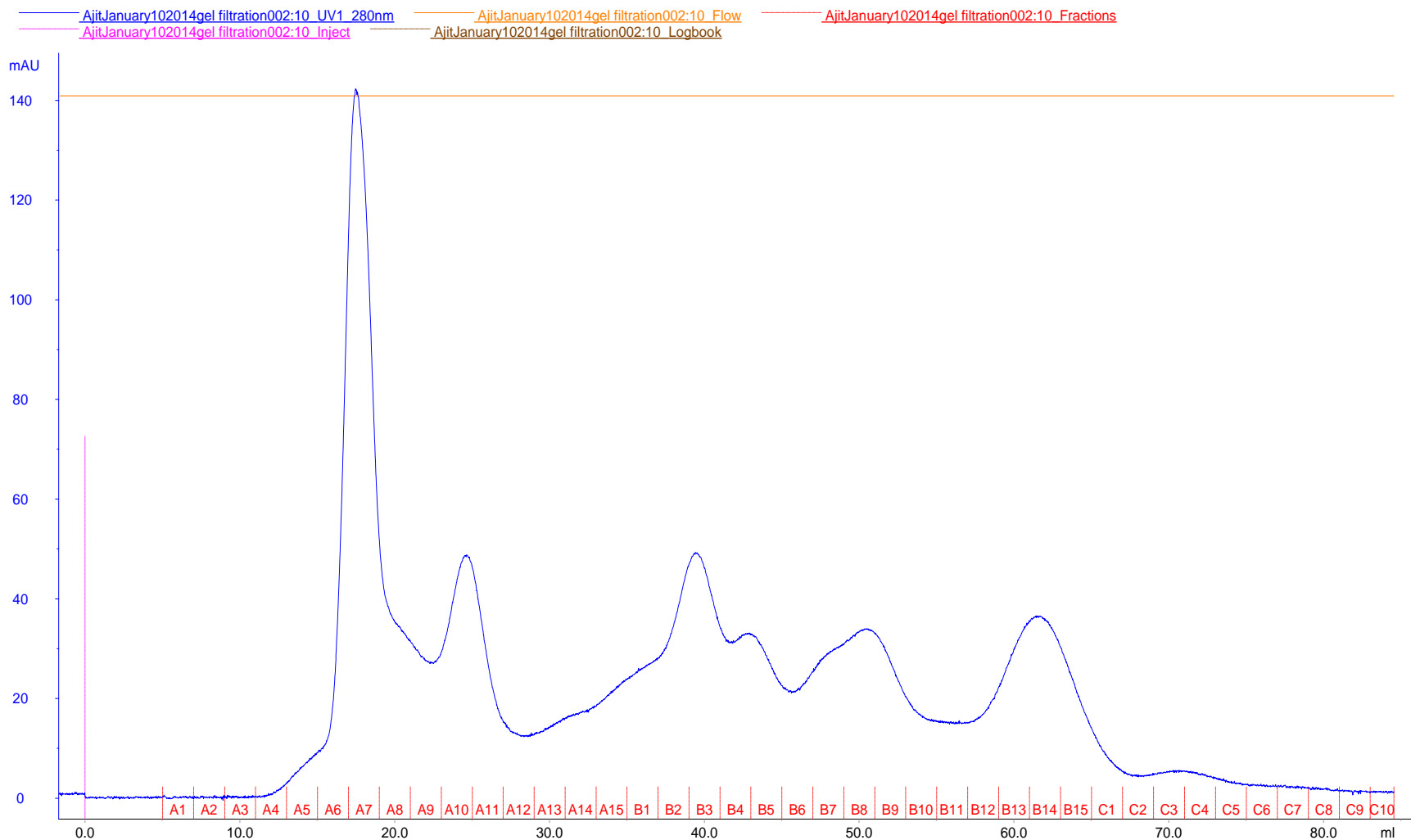

Supplement: Figure S6 — Gel filtration chromatogram for the purification of TfdEI. Fractions A8–A12 hydrolysed cis-dienelactone. (PDF) [file pone.0101801.s006.pdf]

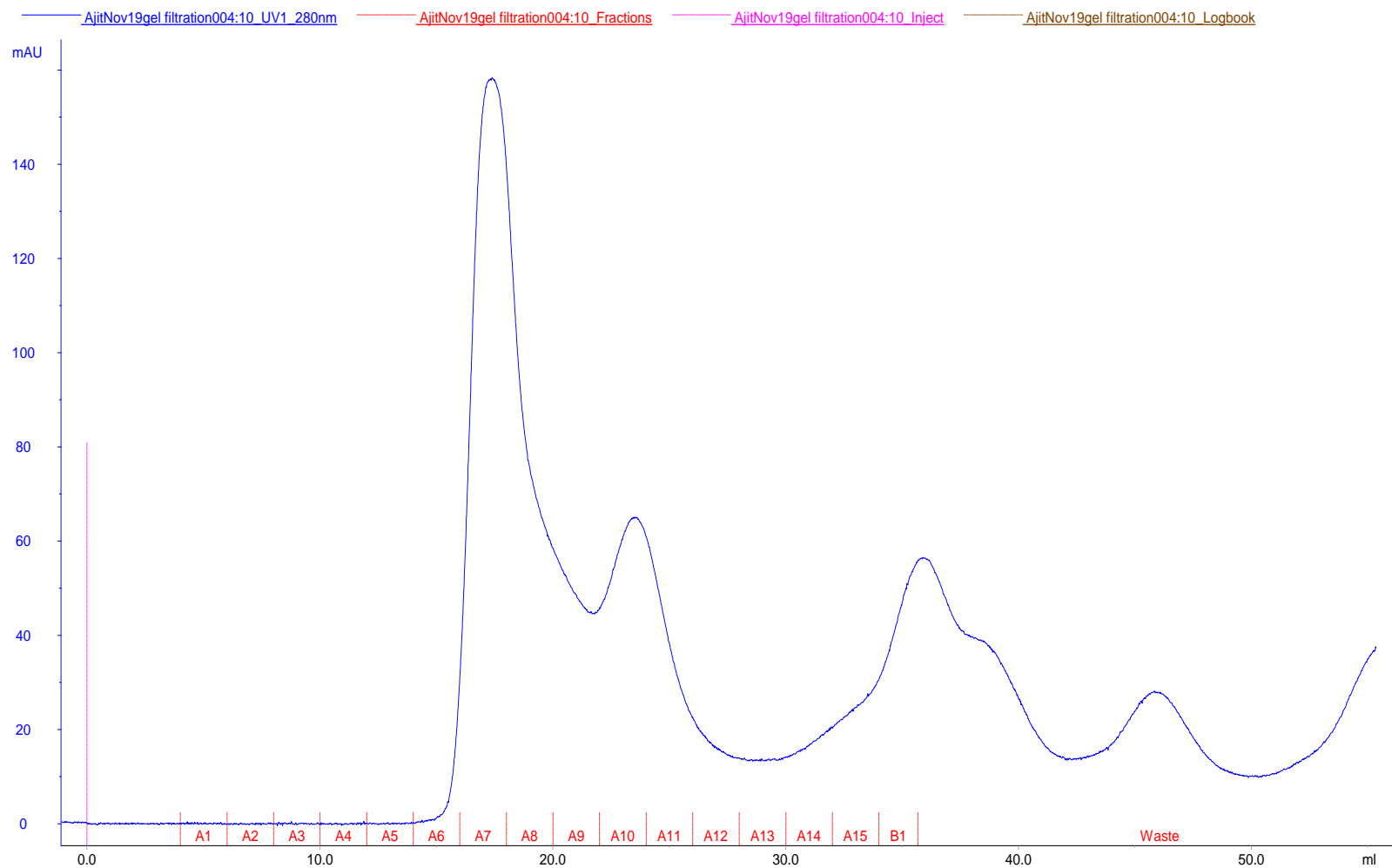

Supplement: Figure S7 — Gel filtration chromatogram for the purification of TfdEII. Fractions A8–A12 hydrolysed cis-dienelactone. (PDF) [file pone.0101801.s007.pdf]
