# Supplementary material for: Two Structurally Different Dienelactone Hydrolases (TfdEI and TfdEII) from Cupriavidus necator JMP134 Plasmid pJP4 Catalyse Cis- and Trans-Dienelactones with Similar Efficiency
Source: PLoS One. 2014 Jul 23;9(7):e101801. doi: 10.1371/journal.pone.0101801 (PMC4108320; doi:10.1371/journal.pone.0101801)
Supplement: Figure S5 — Ion Exchange chromatogram for the purification of TfdEII. Fractions A9–A13 hydrolysed cis-dienelactone. (PDF) [file pone.0101801.s005.pdf]

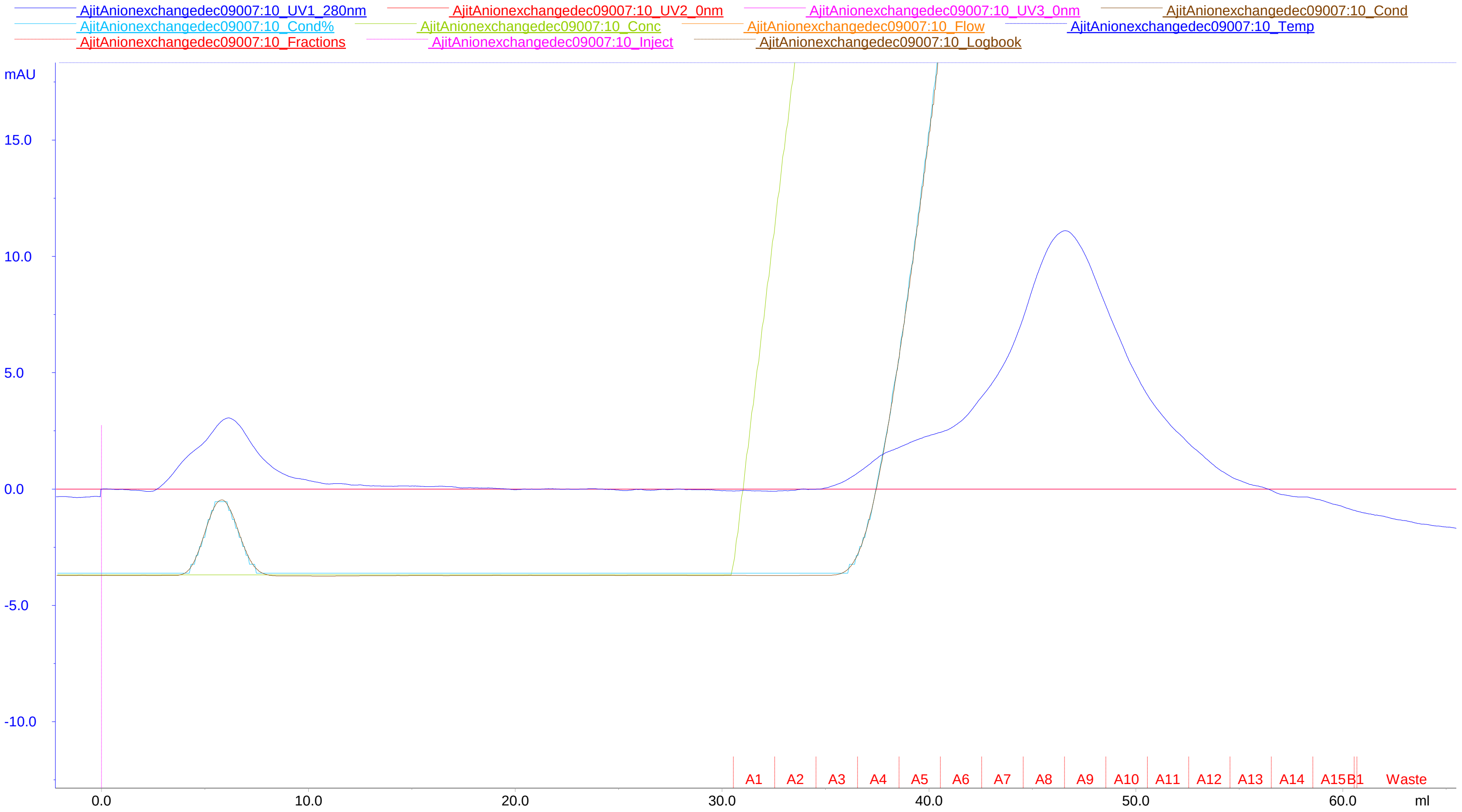

AjitAnionexchangedec09007:10_UV1_280nm
AjitAnionexchangedec09007:10_UV2_0nm
AjitAnionexchangedec09007:10_UV3_0nm
AjitAnionexchangedec09007:10_Cond
AjitAnionexchangedec09007:10_Cond%
AjitAnionexchangedec09007:10_Conc
AjitAnionexchangedec09007:10_Flow
AjitAnionexchangedec09007:10_Temp
AjitAnionexchangedec09007:10_Fractions
AjitAnionexchangedec09007:10_Inject
AjitAnionexchangedec09007:10_Logbook
mAU
15.0
10.0
5.0
0.0
-5.0
-10.0
A1 A2 A3 A4 A5 A6 A7 A8 A9 A10 A11 A12 A13 A14 A15 B1 Waste
0.0 10.0 20.0 30.0 40.0 50.0 60.0 ml
